# Losartan Protects Against Radiation-Induced Testicular Damage by Modulating Oxidative Stress, Testosterone Levels, and Metabolic Profile

**DOI:** 10.3390/ph19010076

**Published:** 2025-12-30

**Authors:** Maria A. Spadella, Rúben J. Moreira, Patrícia C. Braga, Agnaldo B. Chies, Pedro F. Oliveira, Marco G. Alves

**Affiliations:** 1 Human Embryology Laboratory, Marília Medical School, Marilia 17519-030, SP, Brazil; maspadella@gmail.com; 2 Institute of Biomedicine, Department of Medical Science (iBiMED), University of Aveiro, 3010-193 Aveiro, Portugal; rubenjesusmoreira@ua.pt; 3 LAQV-REQUIMTE, Department of Chemistry, University of Aveiro, 3010-193 Aveiro, Portugal; pfobox@gmail.com; 4 Unit for Multidisciplinary Research in Biomedicine (UMIB), Institute of Biomedical Sciences Abel Salazar (ICBAS), 4050-313 Porto, Portugal; patriciacbraga.1096@gmail.com; 5 ITR—Laboratory for Integrative and Translational Research in Population Health, University of Porto, 4050-600 Porto, Portugal; 6 Laboratory of Pharmacology, Marília Medical School, Marilia 17519-030, SP, Brazil; agnaldo.chies.abc@gmail.com

**Keywords:** angiotensin receptor blocker, losartan, radioprotector, radiotherapy, renin-angiotensin system, spermatogenesis

## Abstract

**Background/Objectives:** Testicular dysfunction is a side effect of radiotherapy due to off-target damage. Germ cells are highly vulnerable. Although Sertoli and Leydig cells are more resistant, they are still affected, impairing spermatogenesis and steroidogenesis. With rising youth cancer rates, strategies to preserve fertility are crucial. Losartan (LOS) has potential to mitigate this damage. This work aimed to determine acute and late effects of radiotherapy in testicular metabolism and if LOS mitigates those effects. **Methods:** Male Wistar rats (*n* = 47, 12 weeks old) received 2.5 Gy of ionizing radiation to the scrotum (1.05 Gy/min). LOS-treated rats received 34 mg/kg twice daily before, during and after irradiation. Animals were euthanized at 2 and 60 days post-exposure, to represent acute and late effects, respectively. Reproductive organs were weighed, serum hormones assessed (ELISA), testicular mRNA expression quantified (qPCR) and oxidative stress markers, such as lipid peroxidation, protein carbonylation, and protein nitration measured (slot-blot). Metabolomic profiles were obtained via ^1^H-NMR. **Results:** Acute irradiation reduced seminal vesicle weight, increased FSH, and decreased sperm concentration. Late effects included reduced testicular and epididymal weight, impaired sperm quality, increased protein carbonylation, and altered metabolic profiles. LOS mitigated acute weight loss but not sperm decline. Long-term, LOS improved sperm quality, reduced oxidative stress, and promoted adaptive metabolic responses. **Conclusions:** Irradiation-based cancer therapy causes structural and functional testicular damage and changes the testicular metabolome of rats, while LOS has the potential to be used as a radioprotector to mitigate the adverse acute and late effects of radiation on male fertility.

## 1. Introduction

With the advances in cancer treatment, a growing number of individuals are surviving cancer. Many of these survivors were diagnosed during their reproductive years, a period in which they may consider starting or expanding their families. Radiotherapy, a cornerstone of cancer treatment, utilizes ionizing radiation to precisely target and eliminate tumor cells. While highly effective, it is important to acknowledge that radiotherapy can also unintentionally affect surrounding healthy tissues, including those in the male reproductive system [1]. This off-target damage can lead to a range of long-term consequences, including impaired fertility [2]. Interestingly, a patient survey found that half of male cancer survivors wanted to become fathers in the future [3], have a family, and be a good parent [4]. However, testicular dysfunction after radiation therapy is one of the most problematic sequelae of cancer treatment. Male germ cells, the precursors to sperm, are particularly susceptible to the damaging effects of ionizing radiation. Radiotherapy can disrupt spermatogenesis, the complex and highly regulated process of sperm production, often resulting in male infertility [5]. Indeed, it may have a direct effect on cells or impair the cyclic process of spermatogenesis [6]. Relatively low doses of testicular radiation, above 0.1 Gy, can initiate the impairment of spermatogenesis [7]. In addition, sperm count may not return to its original number following an irradiation dose of 1.2 Gy, in spite of the cases of partial recovery [8]. Furthermore, cumulative doses exceeding 2.5 Gy can lead to irreversible azoospermia, a condition characterized by the absence of sperm in the ejaculate [8]. Higher doses, such as 3.56 Gy, further increase the risk of permanent infertility and hypogonadism, a deficiency in the production of sex hormones [9]. In mice, cumulative low-dose-rate radiation exposure induces ROS production, which leads to a plethora of damages, including sperm DNA damage, lipid peroxidation, and cell senescence, ultimately leading to testicular fibrosis [10]. Furthermore, radiation alters the testicular miRNA profile, especially increasing metabolism-related miRNAs [11].

While it is encouraging that spermatogenesis can recover in some patients following radiotherapy [12], it is important to note that many require assisted reproductive techniques to father biological children. Even in cases of azoospermia or ejaculatory disorders resulting from cancer treatment, sperm retrieval may be possible through specialized procedures like testicular sperm extraction (TESE) [13]. If cancer surgery or radiotherapy causes obstructive azoospermia, sperm can still be retrieved through TESE or microsurgical epididymal sperm aspiration [14]. Importantly, clinical pregnancy and live birth rates in cancer survivors are comparable to those seen in individuals receiving general infertility treatment [15]. However, the risk of genetic damage in descendants conceived from sperm produced during or after radiotherapy remains a topic of ongoing debate and research [16], with the inter- and transgenerational ionizing radiation remaining a topic of discussion [17]. Given the increasing prevalence of cancer diagnoses in infants, juveniles, and young adults, fertility preservation strategies are of paramount importance. Raising awareness among patients, parents, healthcare professionals, and oncologists about the potential impact of cancer treatment on fertility is essential. This will help to ensure that individuals can make informed decisions about their reproductive health and undergo cancer treatment without jeopardizing their future family planning options. Thus, it is still important to understand how radiotherapy affects male reproductive health and which strategies are more suitable to counteract such deleterious effects. Progress in reducing radiotherapy-induced toxicity and in improving selective therapy delivery will translate into better clinical outcomes, lower sperm damage, and earlier recovery of sperm production.

Radioprotection offers a promising avenue for preserving fertility in radiation-exposed men. Losartan (LOS), an angiotensin receptor blocker (ARB) commonly used in clinical practice for the treatment of hypertension, has emerged as a potential radioprotector. The renin–angiotensin system (RAS) is increasingly recognized for its role in radiation-induced cell damage (for review [18]). This effect is suggested to be mediated by the formation of reactive oxygen species (ROS) and apoptosis, triggering cellular damage [19]. In fact, angiotensin II induces the activation of NADPH oxidase, being involved in the production of ROS in organs such as the brain [20] or kidneys [21]. In turn, irradiation increases the expression of RAS [22]. Notably, pharmacological inhibition of RAS, including with LOS, has shown promise in preventing or mitigating these adverse effects associated with cancer treatment in humans and animals [23]. Previous studies have not only demonstrated the presence of RAS in reproductive organs [24] but also the potential for LOS to promote the recovery of seminiferous tubules following radiation exposure [25]. Indeed, rats treated with LOS have a faster recovery of the seminiferous tubules after exposure to 5 Gy irradiation [25]. Our previous work revealed that LOS can mitigate some of the deleterious effects of radiation on testicular function, including improvements in seminal vesicle mass, sperm vitality, and testosterone levels [25]. While somatic testicular cells like Sertoli and Leydig cells, which play supportive roles in spermatogenesis, exhibit greater radioresistance than germ cells, persistent and late-onset effects have been reported [26,27]. Despite the well-established impact of radiotherapy on testicular function, its effects on the testicular metabolome remain largely unexplored. Spermatogenesis is a highly regulated metabolic process, requiring a precise balance of energy production and utilization, as well as intricate signaling pathways [28]. Disruptions to this delicate metabolic system could contribute to infertility. This study uses a metabolomics approach to investigate the hypothesis that exposure to radiation impacts the testicular metabolome short and long-term, and that LOS treatment can mitigate these deleterious metabolic effects. This approach, combined with biometric assessment and oxidative stress evaluation, constitutes a comprehensive approach to assess the radiation-induced testicular damage and putative radioprotective effects of losartan.

## 2. Results

### 2.1. Losartan Prevented Weight Loss in Rats Two Days After Radiotherapy but Not Sperm Quality Decline

**Figure 1:**
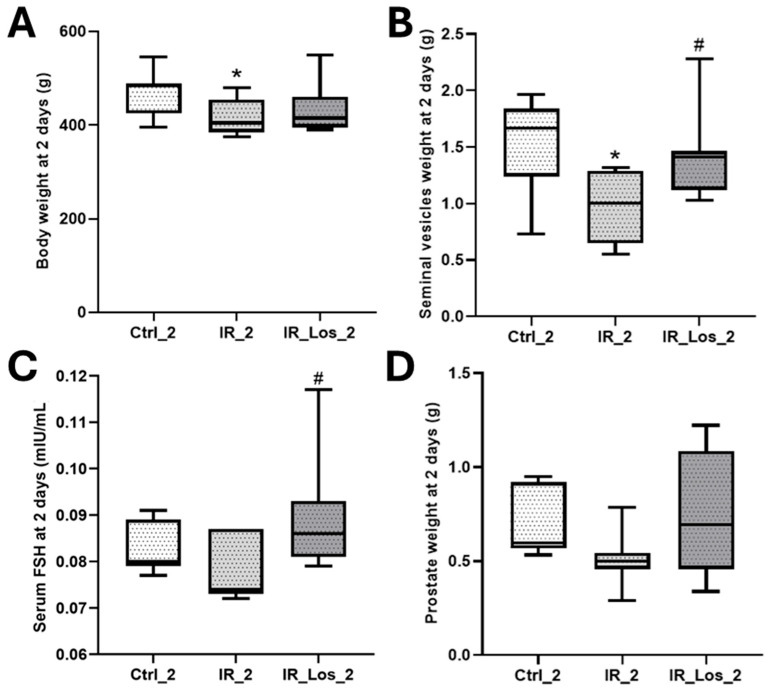
Acute post-irradiation and losartan treatment on biometric parameters in rats. (**A**) body weight, (**B**) seminal vesicle weight, (**C**), serum FSH levels and (**D**) prostate weight in rats 2 days post-irradiation. Ctrl_2, control group at 2 days; IR_2, irradiated group at 2 days; IR_Los_2, irradiated group treated with losartan at 2 days. Data are presented as mean ± standard error of the mean (SEM). * *p* < 0.05 compared to Ctrl_2; # *p* < 0.05 compared to IR_2 (one-way ANOVA followed by Tukey’s post hoc test).

**Figure A1:**
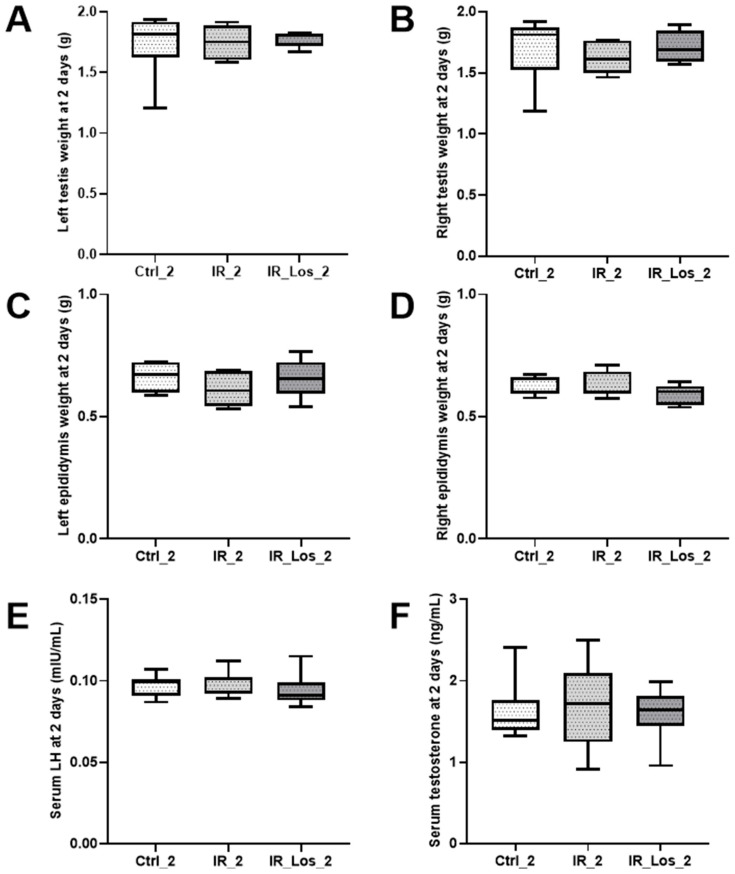
Effect of acute post-irradiation and losartan treatment on testicular and epididymal weights, and serum hormone levels in rats. (**A**) Left testis weight, (**B**) right testis weight, (**C**) left epididymis weight, (**D**) right epididymis weight, (**E**) serum LH levels, and (**F**) serum testosterone levels in rats 2 days post-irradiation. Ctrl_2, control group at 2 days; IR_2, irradiated group at 2 days; IR_Los_2, irradiated group treated with losartan at 2 days. Data are presented as mean ± standard error of the mean (SEM).

**Figure A2:**
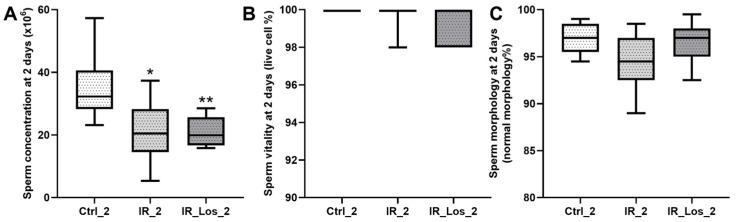
Impact of acute post-irradiation and losartan on sperm parameters in rats. (**A**) Sperm concentration, (**B**) sperm vitality, and (**C**) percentage of sperm with normal morphology in rats 2 days post-irradiation. Ctrl_2, control group at 2 days; IR_2, irradiated group at 2 days; IR_Los_2, irradiated group treated with losartan at 2 days. Data are presented as mean ± standard error of the mean (SEM). * *p* < 0.05, ** *p* < 0.01 compared to Ctrl_2 (one-way ANOVA followed by Tukey’s post hoc test).

Weight loss is one of the most striking deleterious features of external radiotherapy [29]. Acute effects of the radiation exposure (2 days) induced significant weight loss in rats (Figure 1A). Control animals weighed 472.90 ± 18.48 g, while irradiated rats weighed 420.00 ± 15.51 g (*p* < 0.05). This weight loss was accompanied by a significant decrease in seminal vesicle weight (1.51 ± 0.16 g in controls vs. 0.97 ± 0.12 g in irradiated rats, *p* < 0.05, Figure 1B). Notably, there were no alterations in serum FSH levels (0.083 ± 0.002 mIU/mL in controls vs. 0.078 ± 0.003 in irradiated rats, *p* < 0.05, Figure 1C). Although prostate weight also decreased following irradiation (0.69 ± 0.07 g in controls vs. 0.51 ± 0.06 g in irradiated rats, Figure 1D), this difference was not statistically significant. Acute irradiation did not significantly affect testicular weight, epididymal weight, or serum LH and testosterone levels (Figure A1 in Appendix A). Analysis of sperm parameters revealed an acute decrease in sperm concentration following irradiation, while sperm vitality and morphology remained unaffected, as described previously (data presented in Figure A2 in Appendix A) [25]. LOS, an angiotensin receptor blocker (ARB) commonly used in clinical practice, has emerged as a potential radioprotector. LOS effectively prevented radiation-induced weight loss, restoring total body weight to control levels (Figure 1A). LOS treatment also prevented the decrease in seminal vesicle weight observed in irradiated rats (Figure 1B) and increased FSH levels when compared with the levels in irradiated rats (0.079 ± 0.003 mIU/mL vs. 0.090 ± 0.005 mIU/mL, respectively, *p* < 0.05, Figure 1C). As described in previous work, LOS treatment did not prevent the radiation-induced decrease in sperm concentration (data shown in Figure A1 in Appendix A) [25]. LOS treatment in irradiated rats did not significantly affect testicular weight, epididymal weight, or serum LH and testosterone levels (Figure A1 in Appendix A). These findings demonstrate that the acute effects of radiotherapy induce significant weight loss and negatively impact reproductive parameters, specifically seminal vesicle weight and FSH levels. While LOS treatment in irradiated rats effectively mitigates weight loss, it does not fully protect against the detrimental effects of radiation on reproductive function.

### 2.2. Losartan Increased Serum Testosterone in Rats Late Post-Radiotherapy but Did Not Fully Restore Sperm Quality

**Figure 2:**
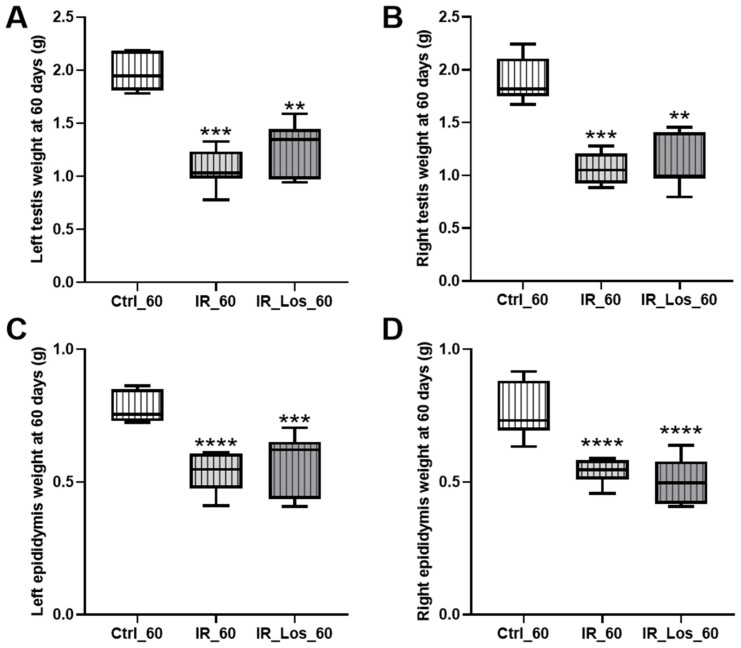
Effects of late post-irradiation and losartan treatment on testicular and epididymal weights in rats. (**A**) Left testis weight, (**B**) right testis weight, (**C**) left epididymis weight, and (**D**) right epididymis weight in rats 60 days post-irradiation. Ctrl_60, control group at 60 days; IR_60, irradiated group at 60 days; IR_Los_60, irradiated group treated with losartan at 60 days. Data are presented as mean ± standard error of the mean (SEM). ** *p* < 0.01, *** *p* < 0.001, **** *p* < 0.0001 compared to Ctrl_60 (one-way ANOVA followed by Tukey’s post hoc test).

**Figure A3:**
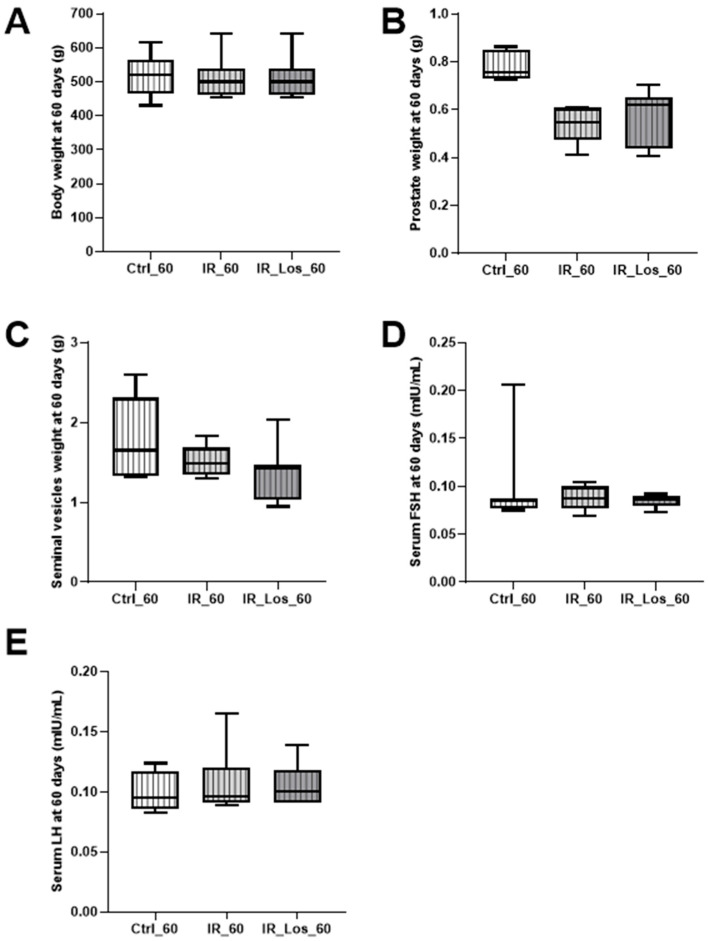
Impact of late post-irradiation and losartan treatment on biometric parameters and serum hormone levels in rats. (**A**) Body weight, (**B**) prostate weight, (**C**) seminal vesicle weight, (**D**) serum FSH levels, (**E**) serum LH levels in rats 60 days post-irradiation. Ctrl_60, control group at 60 days; IR_60, irradiated group at 60 days; IR_Los_60, irradiated group treated with losartan at 60 days. Data are presented as mean ± standard error of the mean (SEM).

Late post-radiotherapy did not induce significant late effects in body weight, prostate weight, or seminal vesicle weight (Figure A3 in Appendix A). Similarly, serum FSH and LH levels remained comparable to those of the control group following long-term post-irradiation (Figure A3 in Appendix A). However, a significant decrease in both left and right testicular weights was observed in irradiated rats (Figure 2A,B). Specifically, left testicular weight decreased from 1.96 ± 0.06 g in controls to 1.06 ± 0.07 g in irradiated rats, and right testicular weight decreased from 1.89 ± 0.08 g in controls to 1.06 ± 0.06 g in irradiated rats (*p* < 0.05 for both). Epididymal weight was also significantly reduced in irradiated rats (Figure 2C,D), with left epididymal weight decreasing from 0.78 ± 0.02 g in controls to 0.53 ± 0.03 g in irradiated rats, and right epididymal weight decreasing from 0.76 ± 0.04 g in controls to 0.54 ± 0.02 g in irradiated rats (*p* < 0.05 for both).

**Figure 3:**
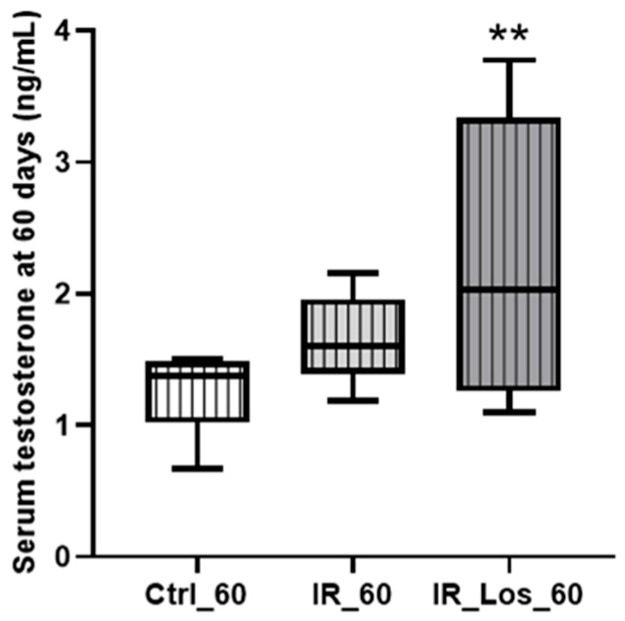
Impact of late post-irradiation and losartan treatment on testosterone levels in rats 60 days post-irradiation. Ctrl_60, control group at 60 days; IR_60, irradiated group at 60 days; IR_Los_60, irradiated group treated with losartan at 60 days. Data are presented as mean ± standard error of the mean (SEM). ** *p* < 0.01 (one-way ANOVA followed by Tukey’s post hoc test).

**Figure A4:**
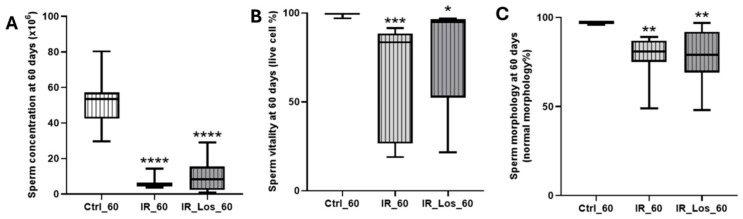
Impact of late post-irradiation and losartan treatment on sperm parameters in rats. (**A**) Sperm concentration, (**B**) sperm vitality, and (**C**) sperm with normal morphology in rats 60 days post-irradiation. Ctrl_60, control group at 60 days; IR_60, irradiated group at 60 days; IR_Los_60, irradiated group treated with losartan at 60 days. Data are presented as mean ± standard error of the mean (SEM). * *p* < 0.05, ** *p* < 0.01, *** *p* < 0.001, **** *p* < 0.0001 compared to Ctrl_60 (one-way ANOVA followed by Tukey’s post hoc test).

While long-term post-irradiation did not significantly affect serum testosterone levels after 60 days (1.25 ± 0.12 ng/mL in controls vs. 1.65 ± 0.13 ng/mL in irradiated rats, Figure 3), treatment with LOS in irradiated rats resulted in a significant increase in serum testosterone compared to controls (2.33 ± 0.40 ng/mL in LOS-treated irradiated rats vs. 1.25 ± 0.12 ng/mL in controls, *p* < 0.05, Figure 3). The late radiation-induced changes in testicular and epididymal weights were accompanied by deleterious effects in sperm quality, which were already described in previous work, along with the protective effects of LOS treatment (data shown in Figure A4 in Appendix A) [25]. LOS treatment did not prevent the radiation-induced decrease in testicular or epididymal weights (Figure 2A–D). Furthermore, LOS treatment did not significantly affect body weight, prostate weight, seminal vesicle weight, or serum FSH and LH levels in irradiated rats (Figure A3 in Appendix A). These findings demonstrate that late post-radiotherapy negatively impacts testicular structure and function, leading to decreased sperm quality, thus inducing late effects. While LOS treatment does not mitigate the effects of radiation on testicular and epididymal weights, it increases testosterone levels and provides a slight improvement in sperm parameters.

### 2.3. Losartan Failed to Mitigate Acute Post-Radiotherapy Testicular Oxidative Stress-Related Damage, but Significantly Reduced Late Effects on Protein Carbonylation in the Testes of Rats

**Figure 4:**
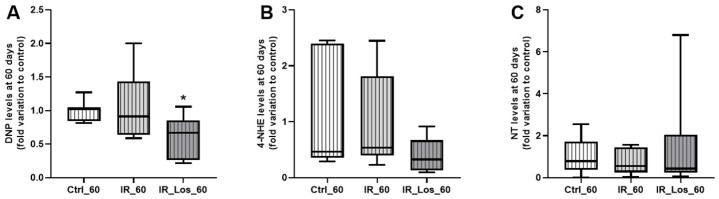
Quantification of oxidative stress markers in rat testes following late post-irradiation and losartan treatment. (**A**) Protein carbonylation (DNP levels), (**B**) lipid peroxidation (4-HNE levels), and (**C**) protein nitration (NT levels) in rat testes 60 days post-irradiation. Ctrl_60, control group at 60 days; IR_60, irradiated group at 60 days; IR_Los_60, irradiated group treated with losartan at 60 days. Data are presented as mean ± standard error of the mean (SEM). * *p* < 0.05 compared to Ctrl_60 (one-way ANOVA followed by Tukey’s post hoc test).

**Figure A5:**
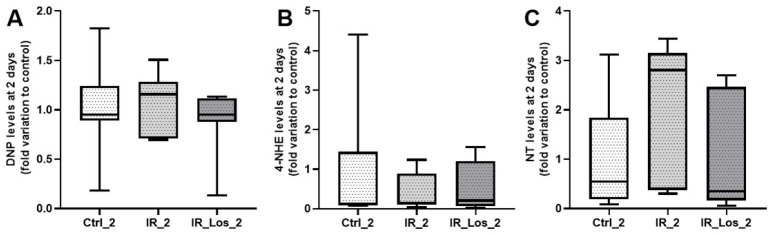
Quantification of oxidative stress markers in rat testes following acute post-irradiation and losartan treatment. (**A**) Protein carbonylation (DNP levels), (**B**) lipid peroxidation (4-HNE levels), and (**C**) protein nitration (NT levels) in rat testes 2 days post-irradiation. Ctrl_2, control group at 2 days; IR_2, irradiated group at 2 days; IR_Los_2, irradiated group treated with losartan at 2 days. Data are presented as mean ± standard error of the mean (SEM).

**Figure A6:**
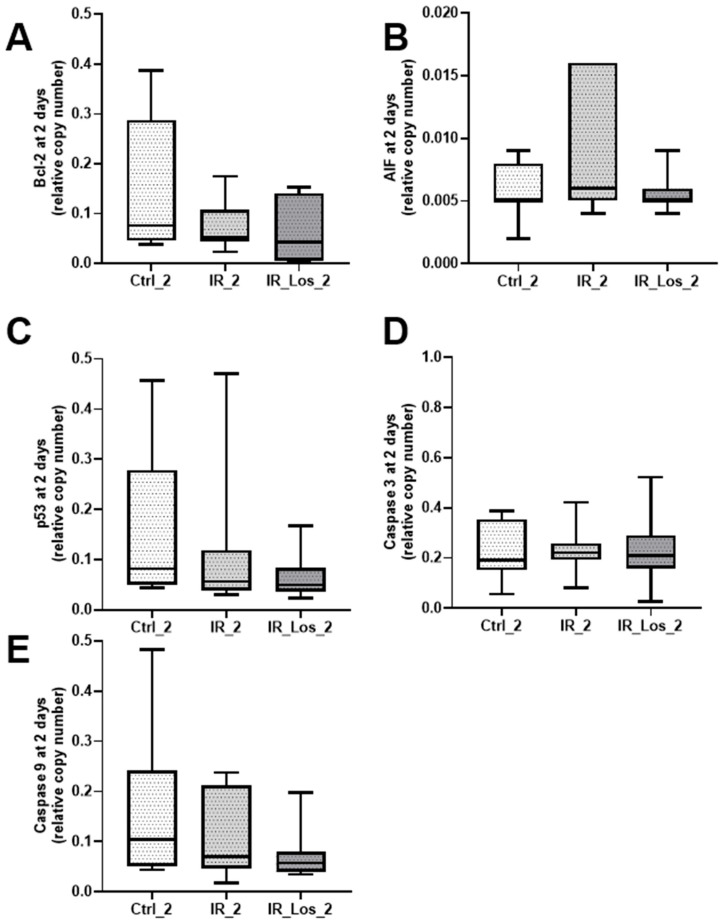
Expression of apoptosis-related proteins in rat testes following acute post-irradiation and losartan treatment. (**A**–**E**) Protein expression levels of (**A**) Bcl-2, (**B**) AIF, (**C**) p53, (**D**) caspase 3, and (**E**) caspase 9 in rat testes 2 days post-irradiation. Ctrl_2, control group at 2 days; IR_2, irradiated group at 2 days; IR_Los_2, irradiated group treated with losartan at 2 days. Data are presented as mean ± standard error of the mean (SEM).

**Figure A7:**
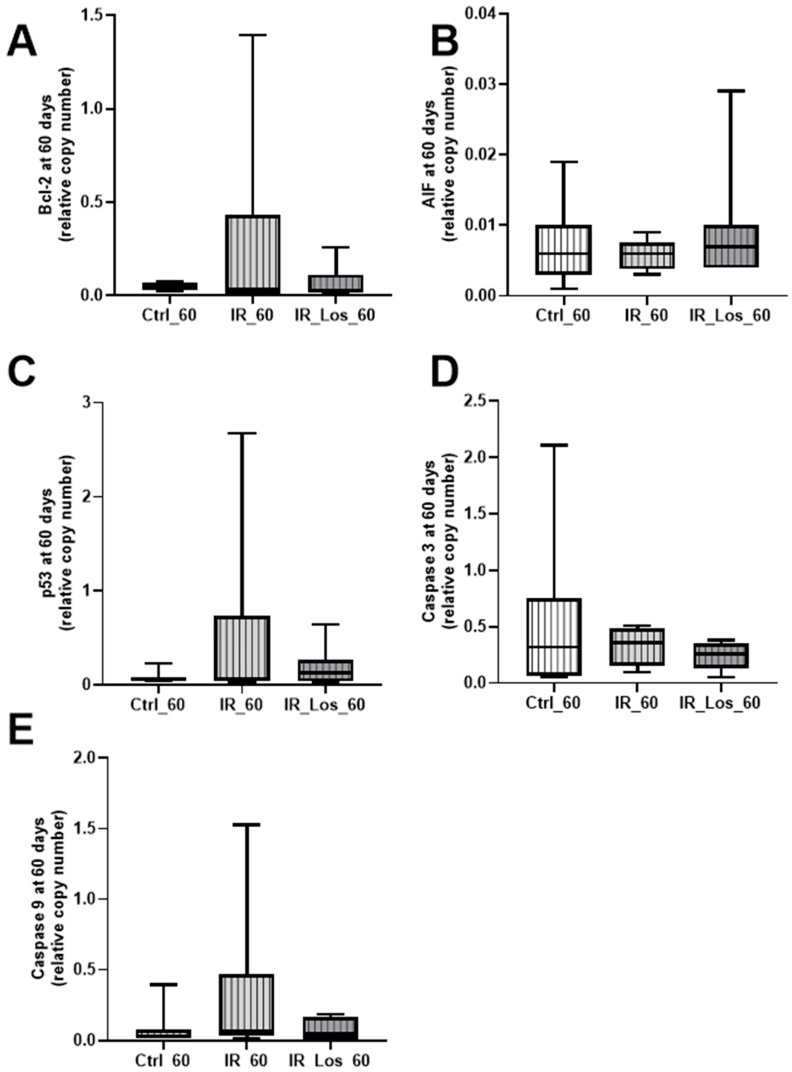
Expression of apoptosis-related proteins in rat testes following late post-irradiation and losartan treatment. Protein expression levels of (**A**) Bcl-2, (**B**) AIF, (**C**) p53, (**D**) caspase 3, and (**E**) caspase 9 in rat testes 60 days post-irradiation. Ctrl_60, control group at 60 days; IR_60, irradiated group at 60 days; IR_Los_60, irradiated group treated with losartan at 60 days. Data are presented as mean ± standard error of the mean (SEM).

Oxidative stress is one of the major deleterious effects of radiotherapy in non-cancerous cells [30]. To assess the impact of radiation and LOS treatment on testicular oxidative stress, we measured protein carbonylation, lipid peroxidation, and protein nitration. Acute post-radiotherapy did not induce significant acute effects in any of these oxidative stress markers, and LOS treatment also had no significant effect on those parameters (Figure A5 in Appendix A). However, long-term post-irradiation resulted in late testicular effects as a slight increase in protein carbonylation (Figure 4A). Specifically, protein carbonylation levels were 1.05 ± 0.22-fold variation to control in irradiated rats compared to 1.00 ± 0.06-fold variation to control in control rats (*p* < 0.05). Long-term post-irradiation did not significantly induce late effects on lipid peroxidation or protein nitration (Figure 4B,C). Notably, LOS treatment significantly attenuated protein carbonylation. Protein carbonylation levels in LOS-treated irradiated rats were 0.63 ± 0.12-fold variation to control, compared to 1.05 ± 0.22-fold variation to control in irradiated rats without LOS treatment (*p* < 0.05, Figure 4A). These findings suggest that long-term, but not short-term post-irradiation, slightly increases oxidative stress in testicular tissue, primarily through protein carbonylation. Furthermore, LOS treatment appears to exert a protective effect against the testis in this specific marker of oxidative damage. To assess the late impact post-irradiation and LOS-treatment on testicular apoptotic pathways, we examined the expression of key apoptosis-related proteins in rat testes. Following acute post-irradiation, no significant changes were observed in the expression of Bcl-2, AIF, p53, caspase 3, or caspase 9 (Figure A6 in Appendix A). Similarly, late post-irradiation did not alter the expression of these proteins, and LOS treatment in late post-irradiated rats had no effect on their expression levels (Figure A7 in Appendix A). These findings suggest that while irradiation may induce some degree of pro-apoptotic signaling, the full apoptotic cascade may not be robustly activated at these time points.

### 2.4. Losartan Mitigates Testicular Metabolic Perturbation Induced by Radiotherapy in a Time-Dependent Manner

**Figure 5:**
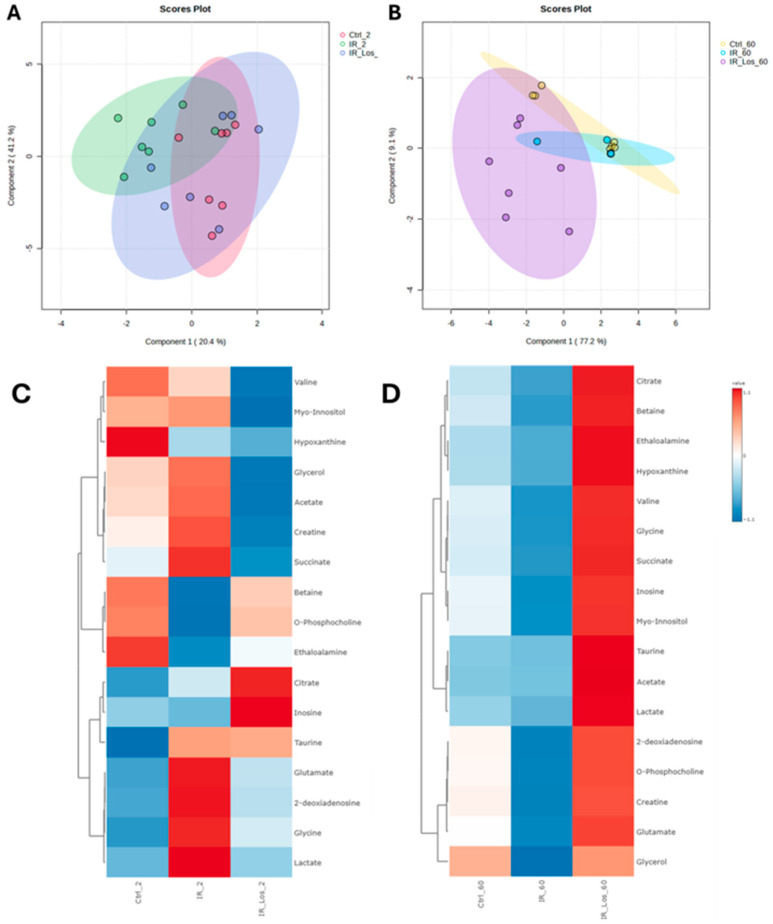
Distinct metabolic profiles of rat testes following irradiation and losartan treatment. sPLS-DA score plots of metabolites identified in rat testes at (**A**) 2 days and (**B**) 60 days post-irradiation. Heatmap analysis of metabolites identified in rat testes at (**C**) 2 days and (**D**) 60 days post-irradiation. Ctrl_2, control group at 2 days; IR_2, irradiated group at 2 days; IR_Los_2, irradiated group treated with losartan at 2 days; Ctrl_60, control group at 60 days; IR_60, irradiated group at 60 days; IR_Los_60, irradiated group treated with losartan at 60 days.

Metabolomic analysis using sPLS-DA and heatmap visualization revealed distinct testicular metabolic profiles between control and irradiated rats at both 2- and 60-days post-exposure, highlighting the impact of acute and late post-irradiation effects on testicular metabolic homeostasis. Interestingly, LOS exerts a significant influence on the metabolic profile of the testes, both in the short and long term. The sPLS-DA score plots show distinct clustering patterns for the different treatment groups. At 2 days post-irradiation, the LOS-treated group (IR_Los_2) exhibits a metabolic profile clearly separate from both the control (Ctrl_2) and irradiated (IR_2) groups (Figure 5A). This suggests a strong and immediate effect of LOS on testicular metabolism. At 60 days, the LOS group (IR_Los_60) still maintains a very distinct profile, particularly from the control group (Ctrl_60) (Figure 5B), suggesting a time-dependent metabolic response to irradiation and a potential persistent metabolic effect of LOS.

**Figure 6:**
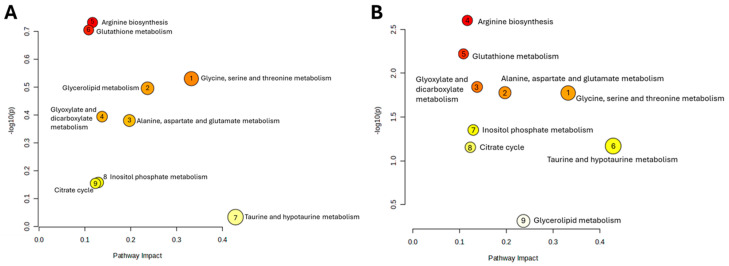
Impact of late post-irradiation and losartan on testicular metabolic pathways. Pathway analysis of metabolites in rat testes 60 days post-irradiation, comparing (**A**) Ctrl_60 vs. IR_60 and (**B**) IR_60 vs. IR_Los_60. Ctrl_60, control group at 60 days; IR_60, irradiated group at 60 days; IR_Los_60, irradiated group treated with losartan at 60 days.

Heatmap visualization further supports these observations. At 2 days post-irradiation, IR_Los_2 displays a unique pattern of metabolite abundance compared to both Ctrl_2 and IR_2 (Figure 5C). At 60 days, the differences are even more pronounced (Figure 5D). These findings suggest that the testis has a metabolic adaptive response to irradiation. Instead, LOS treatment appears to induce a distinct metabolic response in the testes that may be related to its potential protective effects. LOS treatment does not partially mitigate the metabolic alterations induced by acute and late post-radiation effects but may adapt testicular metabolism to promote tissue resilience to radiation-induced damage. Pathway analysis of rat testicular metabolomics data late post-irradiation revealed distinct metabolic alterations in irradiated groups compared to controls. Late post-irradiation significantly impacted pathways in the testis related to amino acid metabolism (glycine, serine, and threonine metabolism; alanine, aspartate, and glutamate metabolism), lipid metabolism (glycerolipid metabolism), and energy metabolism (glyoxylate and dicarboxylate metabolism) compared to the control group (Figure 6A). However, LOS treatment partially reversed those metabolic effects in the testis, restoring glutathione metabolism, inositol phosphate metabolism, and the citrate cycle, suggesting a protective role against irradiation-induced oxidative stress, cellular signaling disruption, and energy imbalance (Figure 6B).

To further investigate the impact of irradiation and LOS treatment in irradiated rats on testicular metabolic pathways, we performed pathway analysis using metabolomics data obtained from rat testicular tissue subjected to late post-irradiation. Table 1 presents the significantly altered pathways in the Ctrl vs. IR comparison, including the pathway name, *p*-value, −log(*p*), false discovery rate (FDR), and pathway impact. As shown in Table 1, late post-irradiation induced significant perturbations in several metabolic pathways within the testes. The most impacted pathways, as indicated by the pathway impact values, included glycine, serine, and threonine metabolism; glycerolipid metabolism; alanine, aspartate, and glutamate metabolism. These pathways are involved in critical testicular functions such as spermatogenesis and steroidogenesis. This suggests that late effects post-irradiation cause widespread disruption of testicular metabolic homeostasis.

Other pathways affected by late effects post-irradiation included purine metabolism, which may affect nucleotide synthesis necessary for DNA replication during spermatogenesis, or galactose metabolism, which could alter energy substrate utilization by sperm. To assess the potential protective effects of LOS on late effects post-irradiation induced metabolic disturbances, we compared the metabolic profiles of irradiated rats (IR) with those treated with LOS (IR_Los) at 60 days post-irradiation (Table 2). LOS treatment significantly modulated several pathways dysregulated by irradiation. Notably, LOS partially or completely restored glutathione metabolism, glycine, serine, and threonine metabolism, alanine, aspartate, and glutamate metabolism. This suggests that LOS may exert protective effects by mitigating the disruption of antioxidant defense, and amino acid metabolism crucial for sperm production, and energy production.

## 3. Discussion

This study investigated the protective effects of LOS, an angiotensin II receptor blocker (ARB), against radiotherapy-induced testicular damage in a rat model. Our findings demonstrate that LOS effectively prevents weight loss following acute post-radiotherapy but does not fully protect against sperm quality decline. In the late post-radiotherapy setting, LOS increases serum testosterone levels but does not fully restore sperm quality or prevent testicular and epididymal weight loss. However, LOS mitigates late effects post-irradiation in protein carbonylation in the testes and demonstrates a time-dependent modulation of testicular metabolic perturbations. These results highlight the complex interplay between radiotherapy, testicular function, and the protective potential of LOS. Our findings confirm that radiotherapy induces significant testicular damage, both acute and late post-irradiation. Acute post-radiotherapy effects were detected in marked weight loss, accompanied by a decrease in seminal vesicle weight and increased serum FSH levels, indicative of impaired reproductive function. This is consistent with previous studies in humans demonstrating the detrimental effects of radiation on the hypothalamic–pituitary–gonadal axis and spermatogenesis [31,32,33,34]. The observed decrease in sperm concentration following acute post-radiotherapy further supports this dysfunction. It is important to note that the impact of radiotherapy on sperm quality depends on several factors, including the dose and duration of irradiation. Our data shows that late post-radiotherapy, while not significantly affecting overall body weight or seminal vesicle weight, led to a significant reduction in testicular and epididymal weights, accompanied by a decline in sperm quality parameters. This suggests that long-term post-radiation may have a more pronounced and prolonged impact on testicular structure and function, potentially leading to long-term infertility [18]. We hypothesized that a radioprotector such as LOS could prevent or at least delay some of the deleterious acute and late post-radiotherapy effects.

LOS has emerged as a potential radioprotector due to its antioxidant, anti-inflammatory, and anti-fibrotic properties [35,36]. RAS is known to play a crucial role in regulating energy balance and metabolism [37]. Polymorphisms in RAS genes, such as the ACE I/D polymorphism, are associated with body weight and adiposity [38,39]. Components of the RAS are expressed locally in adipocytes, and RAS blockade has been shown to induce weight loss in rodents with obesity [40]. It is important to consider energy expenditure, and RAS is likely involved in the modulation of energy balance mainly via the regulation of energy expenditure. Both genetic models of RAS inactivation [41] and diet-induced rodents with obesity treated with ARB [42] exhibit increased energy expenditure independent of energy intake. Finally, there is evidence for the role of RAS in regulating energy intake. Indeed, acute Ang II infusion decreases food intake in rodents [43,44]. Our study revealed that LOS effectively mitigated acute post-radiation effects in weight loss and the associated reduction in seminal vesicle weight. This protective effect may be linked to LOS modulation of the RAS. As previously discussed, the RAS plays a critical role in regulating energy balance and metabolism, and its modulation by LOS may help preserve energy homeostasis in the face of radiation-induced stress [45]. While LOS mitigated the acute post-radiation effects on body weight and seminal vesicle weight, it did not prevent the decline in sperm concentration. This suggests that LOS protective effects on reproductive function in acute post-radiotherapy are limited, particularly regarding spermatogenesis. This is not entirely unexpected, as Leydig cells, which are responsible for steroidogenesis, are generally more radioresistant than the germ cells. Although Leydig cells can be damaged by external irradiation, the doses required for significant dysfunction are typically much higher than those needed to induce germ cell failure [46]. Furthermore, the extent of Leydig cell damage is influenced by both the radiation dose and the age at which the individual is exposed. Higher doses tend to cause more severe damage, while younger individuals, particularly those who are pre-pubertal, are more susceptible to the long-term effects of radiation on Leydig cell function. Most boys who receive 24 Gy for testicular leukemia when they are pubertal or younger are at high risk of delayed sexual maturation associated with decreased testosterone levels and require androgen replacement therapy [47,48]. While most males receiving 20 Gy or less of fractionated testicular irradiation retain normal testosterone production, studies have shown increased plasma LH concentrations in these individuals, suggesting that subclinical injury to Leydig cells may occur even at lower radiation doses [49]. In our late post-radiotherapy effects model, LOS increased serum testosterone levels, which may be associated with the previously described slight improvement in sperm parameters [25]. This finding suggests that LOS may partially preserve Leydig cell function and steroidogenesis, potentially by reducing oxidative stress and inflammation and mediating intratesticular autocrine and paracrine functions [50]. However, it is crucial to note that LOS did not prevent the late post-radiation effects in decreasing testicular and epididymal weights. This indicates that while LOS may offer some protection against functional damage, it does not fully prevent structural damage to the testes caused by long-term post-radiation.

Oxidative stress plays a critical role in the pathogenesis of radiation-induced tissue damage [51]. Our study revealed an interesting distinction between the effects of acute and late post-radiotherapy effects on oxidative stress in the testes. While acute post-radiotherapy did not induce a significant increase in protein carbonylation, a marker of oxidative damage to proteins, late post-radiotherapy did lead to a slight increase. This suggests that late post-exposure to radiation may result in a more sustained accumulation of oxidative damage in the testes. Notably, LOS treatment significantly attenuated protein carbonylation in the testes of rats exposed to late post-radiotherapy. This finding aligns with previous research demonstrating the antioxidant effects of LOS in other models, such as a type 2 diabetes model, where it was shown to decrease carbonylated proteins in the brain [52]. Protein carbonylation is a prevalent and irreversible form of protein oxidation that serves as a reliable marker of oxidative stress. This modification occurs when ROS attacks the side chains of susceptible amino acids, such as lysine, arginine, proline, and threonine, leading to the formation of carbonyl groups. These carbonyl groups can disrupt protein structure and function, ultimately contributing to cellular dysfunction and tissue damage. Our study demonstrated that LOS effectively reduced protein carbonylation in the testes of rats late post-radiotherapy. This protective effect is likely attributed to LOS’s multifaceted antioxidant properties. LOS has been shown to increase the expression of angiotensin-converting enzyme 2 (ACE2), which converts angiotensin II to the antioxidant angiotensin 1–7 [53]. Furthermore, by blocking the action of angiotensin II, a key component of the RAS, LOS can help prevent the overactivation of RAS, which is known to promote ROS production and oxidative stress [54]. In addition to these effects, LOS may also contribute to the restoration of antioxidant enzyme activity, such as catalase and superoxide dismutase, which are essential for scavenging ROS and protecting against oxidative damage [55]. Finally, LOS has been shown to downregulate NADPH oxidase activity, an enzyme complex that generates ROS [56].

Given the intricate link between oxidative stress and tissue metabolism, we conducted a metabolomics analysis to investigate the impact of radiation and LOS treatment on the metabolic profile of the testes. Our metabolomic analysis reveals that both acute and late post-irradiation induce some metabolic disturbances in rat testes. Interestingly, LOS treatment does not simply mitigate these radiation-induced changes but instead elicits an adaptive metabolic response. Both acute and late post-irradiation alter the testicular metabolic profile. We observed significant perturbations in pathways related to amino acid metabolism (glycine, serine, and threonine; alanine, aspartate, and glutamate), lipid metabolism (glycerolipid metabolism), and energy metabolism (glyoxylate and dicarboxylate metabolism). These pathways are intricately linked to critical testicular functions, including spermatogenesis and steroidogenesis, highlighting the potential for irradiation to impair fertility (for review [18]). Late post-irradiation caused widespread disruption of testicular metabolic homeostasis, affecting pathways involved in nucleotide synthesis (purine metabolism) and energy substrate utilization (galactose metabolism), further emphasizing the detrimental impact on sperm production. Contrary to our initial hypothesis, LOS did not simply reverse the irradiation-induced metabolic changes. Instead, both sPLS-DA and heatmap analyses revealed a unique metabolic profile in the LOS-treated groups, distinct from both control and irradiated groups, at both 2- and 60-days post-irradiation. This suggests that LOS actively modifies testicular metabolism, potentially promoting an adaptive response to radiation damage. While LOS did not fully restore the metabolic profile to the control state, it did modulate several key pathways dysregulated by irradiation. Notably, LOS restored glutathione metabolism, crucial for antioxidant defense, and inositol phosphate metabolism, involved in cellular signaling. Additionally, LOS restored the citrate cycle, a central pathway for energy production. These findings suggest that LOS may protect the testes from oxidative stress, cellular signaling disruption, and energy imbalance induced by irradiation [28]. The distinct metabolic profile induced by LOS, coupled with its ability to restore key metabolic pathways, suggests a potential adaptive mechanism that promotes testicular resilience to radiation damage. LOS appears to induce a metabolic state that facilitates long-term testicular adaptation and functional recovery following irradiation. Further research is warranted to elucidate the precise mechanisms underlying LOS metabolic effects and to evaluate its potential as a protective agent against radiation-induced testicular damage. Investigating the long-term impact of LOS on sperm quality, testicular function, and fertility will be crucial in determining its clinical relevance.

This study has some limitations. Firstly, our investigation utilized a rat model. While rodent models provide valuable insights into biological processes, it is important to recognize that the results may not be directly translatable to humans. Further research is needed to determine whether LOS gives similar protective effects in humans. Secondly, we focused on a specific dose and fractionation scheme of radiotherapy, based on our previous work and established dose-dependent effects [18]. While our current findings are generally consistent with our previous work [25], some minor variations in biometric parameters, such as weight, were observed. These differences likely reflect inherent biological variability between animals, including differing responses to both irradiation and LOS treatment. For example, the efficacy of LOS-mediated radioprotection may vary depending on individual animal responses and potentially require different treatment durations. Additionally, the presence of post-irradiation diarrhea in some animals but not others further highlights this individual variability. Given the potential variability in response to different radiation regimens, future studies should explore the effects of LOS under a wider range of radiotherapy conditions. Finally, this study did not investigate the long-term consequences of radiation-induced testicular damage on fertility outcomes. It is possible that the protective effects of LOS on sperm parameters may translate into improved long-term fertility. Future studies should include long-term assessments of fertility parameters to fully evaluate the clinical significance of LOS protective effects. Despite these limitations, our findings provide a foundation for future research aimed at elucidating the complex interplay between radiotherapy, testicular function, and the protective potential of LOS. Further studies should address the limitations outlined above and delve deeper into the underlying mechanisms by which LOS exerts its protective effects on testicular function after radiotherapy. This includes investigating the specific molecular pathways involved, the impact of LOS on different cell types within the testes, and the potential for combination therapies with other radioprotective agents.

## 4. Materials and Methods

### 4.1. Animal Model and Experimental Design

Forty-seven 12-week-old male Wistar rats weighing between 350 and 400 g were obtained from the Central Vivarium of Marília Medical School (FAMEMA), Marília, São Paulo State, Brazil. The animals were maintained in polypropylene cages (50 cm × 40 cm × 20 cm; 3 animals/cage), under a 12 h light/12 h dark cycle at a controlled temperature (23 °C ± 1 °C), with free access to water and pelleted rodent chow. These housing conditions were strictly maintained and constant throughout the experimental design and the personnel responsible for animal monitoring were blinded to the allocation of each animal to the experimental groups.

**Figure 7:**
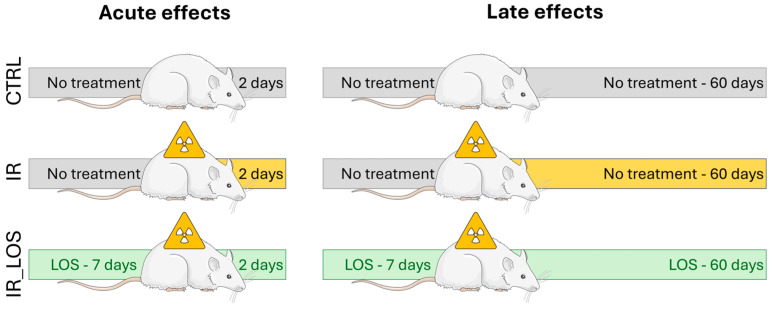
Schematic representation of the experimental design, showing the animal model (Wistar rats) and time points of exposure to radiation and losartan treatment.

This study was approved by the Ethics Committee for the Use of Experimental Animals from the Marília Medical School (CEUA/FAMEMA, protocol number 1525/19) and conducted according to the National Council of Animal Experimentation Control (CONCEA, Brazil). The experiments were performed in two distinct timepoints (Figure 7): (1) 2 days post-testicular irradiation to assess the acute effects and (2) 60 days post-testicular irradiation to assess late damage, considering a constant 58 day-spermatogenic cycle in rats [57]. The rats were randomly separated in six experimental groups (*n* = 8 per group, except IR_60, *n* = 7): Ctrl_2 (control), non-irradiated and non-treated, euthanized after 2 days post-irradiation; IR_2, irradiated and non-treated, euthanized 2 days after irradiation; IR_Los_2, irradiated and treated with losartan, euthanized 2 days after irradiation; Ctrl_60 (control), non-irradiated and non-treated, euthanized after 60 days post-irradiation; IR_60, irradiated and non-treated, euthanized 60 days after irradiation; IR_Los_60, irradiated and treated with losartan, euthanized 60 days after irradiation. The protocol for radiation was based on our previous studies [25,58]. In brief, rats were weighed, anesthetized with tribromoethanol (25 mg/100 g; i.p.; Sigma-Aldrich, MO, USA), and immobilized on a wooden support. The scrotum was gently retracted and secured. To ensure uniform irradiation dose delivery to the testes, the scrotum was positioned within a paraffin-filled casing. A single dose of 2.5 Gy of ionizing radiation was delivered to the scrotal area at a dose rate of 1.05 Gy/min using a Varian Clinic 6EX linear accelerator (Varian Medical Systems, Palo Alto, CA, USA) operating at 6 MV. The irradiation was administered at a source-to-surface distance (SSD) of 100 cm, with a 5 × 5 cm radiation field encompassing the scrotum in the anteroposterior direction. The animals were irradiated in the supine position at a depth of 2.0 cm. Control animals underwent the same anesthesia, immobilization, and scrotal manipulation. The selected radiation dose was based on our previous studies that included a dose–response curve for testicular effects in rats [25,58]. The rats treated with LOS (Gemini, Goiás, Brazil) received 34 mg/kg twice per day, administered by gavage. Before each administration, 0.5% LOS solution was diluted in carboxymethylcellulose (Denver Especialidades Químicas Ltd., São Paulo, Brazil). The treatment with LOS started 7 consecutive days prior to irradiation, maintained during the day of exposure, and continued for either 2 or 60 consecutive days to assess its effects on acute and late testicular damage, respectively. Based on previous studies on rats, this LOS dosage does not cause gonadotoxicity [25,59]. The non-LOS-treated animals received only the vehicle twice daily.

### 4.2. Biometric and Sperm Quality Characterization

At 2- or 60-days post-irradiation, rats were weighed and euthanized via CO_2_ inhalation followed by exsanguination. Blood samples were collected via inferior vena cava puncture using BD Vacutainer^®^ blood collection tubes (Becton, Dickinson and Company, Franklin Lakes, NJ, USA) for subsequent analysis of FSH, LH, and testosterone concentrations. Collected blood samples were centrifuged at 1917× *g* for 20 min at 4 °C to separate plasma, which was then stored at −80 °C until hormonal assays were performed. The testes, epididymides, prostate, and seminal vesicles were excised and weighed. Organ wet weights (g) were normalized to the respective body weight (kg) of each animal. Serum FSH/LH and Plasma testosterone levels were quantified by enzyme-linked immunosorbent assay (ELISA; FSH [RE52121, analytical sensitivity 0.86 mlU/mL], LH [RE52101, analytical sensitivity 1.27 mlU/mL], and testosterone [RE52151, analytical sensitivity 0.12 ng/mL]; IBL International, Hamburg, Germany) according to the manufacturer’s instructions. The sperm parameters were analyzed and previously reported [25].

Sperm vitality was assessed using an eosin-nigrosine staining technique Briefly, 50 μL of the sperm suspension was mixed with one drop of 3% eosin-Y, followed by two drops of 8% nigrosine after 3 min. The percentages of live (unstained) and dead (stained) sperm were determined by counting 200 spermatozoa. Sperm morphology was evaluated by preparing smears with 10 μL of the sperm suspension, staining with Shorr stain and hematoxylin, and observing under a light microscope (1000x magnification). A total of 200 spermatozoa were assessed per sample and classified as normal or abnormal according to the criteria described by Filler [25].

### 4.3. Polymerase Chain Reaction

Total DNA and RNA extractions from testicular tissue were performed using an NZY Tissue gDNA Isolation Kit and an NZY Total RNA Isolation Kit, respectively, as per the manufacturer’s instructions. The extracted DNA and RNA were quantified using a NanoDrop One Spectrophotometer (Thermo Fisher Scientific, Waltham, MA, USA). cDNA was obtained from extracted total RNA using NZY M-MuLV Reverse Transcriptase. Specific cDNA fragments were amplified using a designed exon–exon spanning primer set (Table 3), with optimal annealing temperatures. B-cell lymphoma 2 (Bcl-2), Apoptosis-inducing factor (AIF), cellular tumor antigen p53 (p53), Caspase 9, and Caspase 3 mRNA levels were evaluated in testicular tissue, and β-2-microglobulin (β2M) transcript levels were used to normalize expression. Target genes, sequences and annealing temperatures are presented in Table 3. Amplification and quantitative PCR (qPCR) experiments were carried out with a CFX Duet Real-time PCR system (Biorad, Hercules, CA, USA). The fold variation in the expression of target testicular genes was calculated using the mathematical model suggested by Pfaffl [60] using the formula: 2^−∆Ct^, where ∆Ct corresponds to the deviation of the control sample from the reference of the transcript gene.

### 4.4. Determination of Oxidative Stress-Related Markers

Lipid peroxidation, protein carbonylation, and protein nitration were assessed by immunoblotting using a slot-blot system. For lipid peroxidation and protein nitration, 10 µg of protein was diluted in PBS to 100 µL and transferred to nitrocellulose membranes. For protein carbonylation, 5 µg of protein was diluted in PBS to 20 µL, then 20 µL of 12% SDS and 40 µL of 20 mM DNPH in 10% TFA were added for derivatization (30 min, room temperature, dark). The reaction was stopped with 30 µL of 2 M Tris/18% β-mercaptoethanol, and 2.4 µL of the sample was diluted in 107.6 µL PBS and transferred to PVDF membranes. All membranes were blocked with 5% non-fat milk in TBS-T for 1 h and incubated overnight with primary antibodies against 4-HNE (1:1000; AB5605, Sigma-Aldrich), Nitro-Tyrosine (1:1000; 9691S, Cell Signaling, Danvers, MA, USA), or DNP (1:5000; D9656-2ML, Sigma-Aldrich, St. Louis, MO, USA). After washing, membranes were incubated with anti-goat (A4187, Sigma-Aldrich, St. Louis, MO, USA) or anti-rabbit (AB6721, Abcam, Cambridge, UK) secondary antibodies (1:1000 in 1% non-fat milk TBS-T) for 1 h. Immuno-reactive proteins were detected with ECL substrate (BIO-RAD, Hercules, CA, USA) on a Bio-Rad ChemiDoc system. The band densities were quantified using Image Lab™ 6.0 Software (Bio-Rad, Hercules, CA, USA) and normalized to the control.

### 4.5. Determination of Testis Metabolome

For testicular metabolite extraction, tissue was homogenized in a 2:1 methanol: chloroform mixture and sonicated on ice for 15 min. Following the addition of chloroform and water (1:1), samples were centrifuged at 10,000× *g* for 15 min at 4 °C. The resulting polar and apolar fractions were isolated and evaporated under a nitrogen stream. The polar fraction was then reconstituted in 0.2 M phosphate buffer (pH 7) in D_2_O for ^1^H-NMR analysis. ^1^H-NMR spectra were acquired on a Bruker Avance III HD 500 MHz spectrometer (Bruker Corporation, Billerica, MA, USA) equipped with a 5 mm TXI probe. Solvent-suppressed spectra were obtained at 298K using a NOESYPR1D pulse sequence with the following parameters: sweep width = 7 kHz, relaxation delay = 7 s, pulse angle = 30°, acquisition time = 2.3 s, and 128 scans. Free induction decays were multiplied by a 0.2 Hz Lorentzian prior to Fourier transformation. 480 μL of each sample was analyzed after dilution with sodium fumarate (2 mM final concentration) in deuterated water, which served as an internal reference, to a final volume of 600 μL. Spectra were manually phased and baseline-corrected, and peaks were assigned using Chenomx NMR Suite 12.0 software (Chenomx Inc., Edmonton, AB, Canada) and the Human Metabolome Database (HMDB). Spectra analysis and metabolite quantification were performed using NUTS-Pro NMR software (https://www.emory.edu/NMR/docs/nuts_manual.pdf, accessed on 18 December 2025) (Acorn NMR, Inc., Fremont, CA, USA). The following metabolites were identified in the testicular tissue (multiplet, chemical shift (ppm)): valine (doublet, 0.98), myo-inositol (multiplet, 3.54), hypoxanthine (singlet, 8.19), glycerol (multiplet, 3.55), acetate (singlet, 1.92), creatine (singlet, 3.94), succinate (singlet, 2.39), betaine (singlet, 3.25), O-phosphocholine (singlet, 3.20), ethanolamine (triplet, 3.36), citrate (multiplet, 2.50), inosine (singlet, 8.19), taurine (triplet, 3.26), glutamate (multiplet,3.75), 2-deoxyadenosine (singlet, 8.33), glycine (singlet, 3.54), lactate (doublet, 1.31). Results are presented as nmol/mg of dry protein.

### 4.6. Statistical Analysis

Multivariate analysis was performed using MetaboAnalyst 6.0 (https://metaboanalyst.ca/, accessed on 20 November 2024). Briefly, data were log-transformed and auto-scaled prior to principal component analysis (PCA) and partial least squares discriminant analysis (PLS-DA). Variables with variable importance in projection (VIP) scores > 1 were considered significant for cluster separation. Model quality was assessed by 5-fold cross-validation, with R2 and Q2 values indicating observed variation and predictive potential, respectively. Univariate analysis was conducted using GraphPad Prism 9.5 (La Jolla, CA, USA) and IBM SPSS Statistics 29.0 (Chicago, IL, USA). Data normality was assessed with the Shapiro–Wilk test. Differences in testicular metabolite concentrations, biometric parameters, qPCR, and slot blot data between experimental groups were evaluated using one-way ANOVA for multiple comparisons and Student’s unpaired *t*-tests for pairwise comparisons, both with a false discovery rate (FDR) of 0.05. In the text, the data are presented as mean ± standard error of the mean (SEM) of replicates of *n* = 8 (except for IR_60, which was *n* = 7).

## 5. Conclusions

In conclusion, our study provides evidence that LOS partially protects against radiotherapy-induced testicular damage in rats. While LOS effectively prevents weight loss after acute post-radiotherapy, it does not fully protect against sperm quality decline. In the late post-radiotherapy setting, LOS increases serum testosterone levels and attenuates protein carbonylation in the testes but does not fully restore sperm quality or prevent testicular and epididymal weight loss. LOS also demonstrates a modulation of testicular metabolic perturbations, suggesting a potential adaptive response towards metabolic homeostasis and promoting resilience to radiation-induced damage. These findings contribute to our understanding of the complex interplay between radiotherapy, testicular function, and the protective potential of LOS, paving the way for future research aimed at developing effective strategies to mitigate the adverse effects of radiation on male fertility. In the long term, these findings suggest the potential repurposing of LOS as a novel radioprotective agent, with putative benefits including the reduction in oxidative stress and the increase in serum testosterone. Further clinical evidence is required to fully validate the efficacy of this strategy.

## Figures and Tables

**Table 1:** Significantly altered pathways identified by metabolomics analysis in irradiated (IR_60) rats compared to control (Ctrl) rats 60 days after irradiation. The table includes pathway name, *p*-value, −log(*p*), false discovery rate (FDR), and pathway impact score.

Pathway Name	*p*	−log(*p*)	FDR	Impact
Arginine biosynthesis	0.18561	0.7314	0.56164	0.11675
Glutathione metabolism	0.1975	0.70443	0.56164	0.10839
Glycine, serine and threonine metabolism	0.29519	0.5299	0.63904	0.3323
Glycerolipid metabolism	0.31952	0.4955	0.63904	0.23676
Glyoxylate and dicarboxylate metabolism	0.40388	0.39375	0.67083	0.13757
Alanine, aspartate and glutamate metabolism	0.41706	0.3798	0.67083	0.19712
Inositol phosphate metabolism	0.69537	0.15778	0.82811	0.12939
Citrate cycle (TCA cycle)	0.70071	0.15446	0.82811	0.12311
Taurine and hypotaurine metabolism	0.92575	0.033507	0.94219	0.42857

**Table 2:** Significantly altered pathways identified by metabolomics analysis in irradiated (IR_60) rats compared to irradiated rats treated with LOS (IR_Los_60) at 60 days post-irradiation control (Ctrl) rats 60 days after irradiation. The table includes pathway name, *p*-value, −log(*p*), false discovery rate (FDR), and pathway impact score.

Pathway Name	*p*	−log(*p*)	FDR	Impact
Arginine biosynthesis	0.0024959	2.6028	0.021631	0.11675
Glutathione metabolism	0.0060079	2.2213	0.026034	0.10839
Glyoxylate and dicarboxylate metabolism	0.014336	1.8436	0.033617	0.13757
Alanine, aspartate and glutamate metabolism	0.016675	1.7779	0.033617	0.19712
Glycine, serine and threonine metabolism	0.016809	1.7745	0.033617	0.3323
Inositol phosphate metabolism	0.044451	1.3521	0.055034	0.12939
Taurine and hypotaurine metabolism	0.067794	1.1688	0.079131	0.42857
Citrate cycle (TCA cycle)	0.070001	1.1549	0.079131	0.12311
Glycerolipid metabolism	0.48687	0.31259	0.48687	0.23676

**Table 3:** Primer sequences used for quantitative real-time PCR analysis. The table lists the target genes, primer sequences (5′-3′), annealing temperatures (AT), and cycle numbers used for quantitative real-time PCR (qPCR) analysis.

Gene	Primer Sequence (5′-3′)	AT (°C)	Cycles
p53	Sense: CTGCCCACCACAGCGACAGG Anti-sense: AGGAGCCAGGCCGTCACCAT	60	35
Casp-3	Sense: AGGCCTGCCGAGGTACAGAGC Anti-Sense: CCGTGGCCACCTTCCGCTTA	60	35
Casp-9	Sense: TGCAGGGTACGCCTTGTGCG Anti-Sense: CCTGATCCCGCCGAGACCCA	60	35
AIF	Sense: CGGCGGTGTGTGAAAAGAAA Anti-Sense: ATTTTGCCCCCTGATGGACC	56	30
BCL-2	Sense: GGTGAACTGGGGGAGGATTG Anti-Sense: AGAGCGATGTTGTCCACCAG	58	30
β2-M	Sense: AGGTTTGAGGGGGAATGCTG Anti-sense: ATGAGTATGCCTGCCGTGTG	58	35

Abbreviations: *Casp-3*—caspase 3; caspase 9; *AIF*—Apoptosis-Inducing Factor; *BCL-2*—B-cell lymphoma 2; *β2-M*—beta-2 microglobulin.

## Data Availability

Dataset available on request from the authors.
